# The Choice of Rhodium Catalysts in [2+2+2] Cycloaddition Reaction: A Personal Account

**DOI:** 10.3390/molecules27041332

**Published:** 2022-02-16

**Authors:** Anna Pla-Quintana, Anna Roglans

**Affiliations:** Institut de Química Computacional i Catàlisi (IQCC) and Departament de Química, Universitat de Girona (UdG), Facultat de Ciències, C/Maria Aurèlia Capmany, 69, 17003 Girona, Catalunya, Spain

**Keywords:** rhodium, catalysis, [2+2+2] cycloadditions

## Abstract

[2+2+2] Cycloaddition reaction is a captivating process that assembles six-membered rings from three unsaturations with complete atom economy. Of the multiple transition metals that can be used to catalyze this reaction, rhodium offers many advantages. These include high activity and versatility, but especially the ability to easily tune the reactivity and selectivity by the modification of the ligands around the metal. In this personal account, we summarize our endeavours in the development of efficient and sustainable [2+2+2] cycloaddition reactions to prepare products of interest, develop conditions in which the catalyst can be recovered and reused, and understand the mechanistic details that govern the selectivity of the processes.

## 1. Introduction

**Figure 1:**
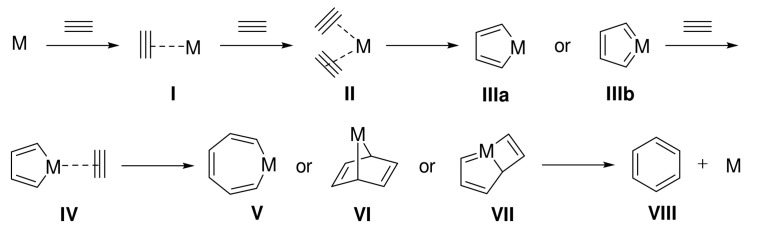
The mechanistic proposal for [2+2+2] cycloaddition reactions of three alkynes. Reprinted with permission from [3]. Copyright 2012 John Wiley and Sons.

The use of transition-metal catalysts to perform [2+2+2] cycloaddition reactions of unsaturated substrates remains an attractive approach for the construction of cyclic skeletons from the perspective of atom and step economy. In 1948, Reppe et al. [1] described the first transition metal-catalysed [2+2+2] cycloaddition of three alkynes under nickel catalysis to obtain substituted benzene derivatives. Since this first study, many examples of alkyne cycloaddition catalysed by different transition metals have been described. The metals which have been used most are Ni, Co, Pd, Rh, Ru, Zr, and Ir. In addition to alkynes, the participation of a wide range of other unsaturations, such as alkenes, allenes, and nitriles, in these cycloadditions enables the construction of a large number of six-membered carbo- and heterocyclic compounds with a high degree of functionality. The fine-tuning of the metal/ligand combinations and the reaction conditions allows chemo-, regio-, diastereo-, and enantioselectivity to be achieved in these transformations [2]. The generally accepted mechanism for this process entails an oxidative coupling of two alkynes to generate a metallacyclopentadiene intermediate into which the third alkyne is inserted. Finally, the arene is formed by the reductive elimination of the metal (Figure 1).

**Scheme 1:**
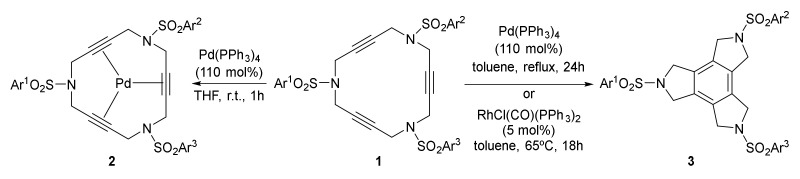
The reaction of triacetylenic azamacrocycles **1** with Pd(0) complexes.

In 2004, we were involved in a project based on the design and synthesis of new palladium complexes with azamacrocycles, containing double bonds in their structure, and their applications in catalysis [4,5]. In particular, 15-membered triolefinic azamacrocyles were shown to be excellent ligands for Pd(0) through the three double bonds and were used as recoverable and reusable catalysts in Heck reactions and Suzuki–Miyaura cross-couplings using diazonium salts [6,7,8,9]. With the idea of further exploring these azamacrocycles as ligands for palladium, we extended the complexation studies to 15-membered triacetylenic azamacrocycles of type **1** (Scheme 1). In this case, we found different final products depending on the reaction conditions. Macrocycle **1** formed a stable complex with Pd(0), **2**, when it was treated with Pd(PPh_3_)_4_ at room temperature. However, compound **1** underwent a [2+2+2] cycloaddition reaction converting it into the corresponding triazatrindane **3**, when treated with Pd(PPh_3_)_4_ in refluxing toluene [10] (Scheme 1). Unfortunately, the cycloisomerization reaction did not proceed when only catalytic quantities of the palladium complex were used. In order to avoid the stoichiometric quantities of palladium, other transition metals were tested and, of these, the Wilkinson’s complex imparted the best performance.

At this point, we noticed that we had an interesting chemistry on our hands, and this was our starting point for a research line that has yielded relevant new results for the last 15 years. We have developed cyclization reactions using rhodium as the transition metal and have tackled several different aspects of the chemistry: the methodologic development of intra- and intermolecular cycloaddition reactions, including enantioselective versions; the participation of unsaturations other than alkynes such as alkenes, nitriles, and allenes; the development of heterogeneous catalysts; mechanistic studies by DFT calculations and experimental techniques; and the application to the synthesis of compounds with potential value in the field of materials.

In this personal account, we would like to show how the efficiency and success of our cycloaddition reactions has depended mainly on the different rhodium-based catalytic systems that we have used. Although we started with the classical Wilkinson’s catalyst, we have also widely explored many combinations of cationic rhodium complexes with different bisphosphine ligands, as well as rhodium complexes with iminophosphoranes, phosphoramidite, and carbene ligands. Given this fact, we have organized this account around the Rh catalytic system that we have used throughout all of our studies, with the goal of providing a guide for selecting the best catalyst for a given reaction.

## 2. Results

### 2.1. Wilkinson’s Catalyst

**Scheme 2:**
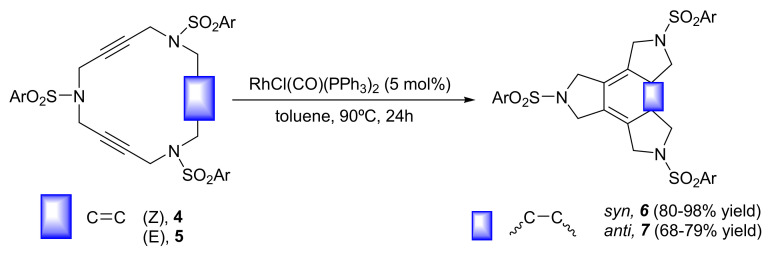
[2+2+2] The cycloaddition reactions of cyclic enediynes **4** and **5**.

As we have stated in the introduction, the first challenge that we faced in this research line was finding a catalytic system that promoted the [2+2+2] cycloaddition of triacetylenic macrocycles **1** (Scheme 1) in an efficient way. Among the transition metals that promote this cycloaddition, cobalt, ruthenium, and rhodium are three of the most widely used [2]. We started by testing CpCoL_2_ systems, since their activity in this kind of processes had been previously demonstrated by Vollhardt and Bergman [11,12,13]. Catalytic amounts of CpCo(CO)_2_ (5% mol) in n-decane at 140 °C only delivered a 44% yield of cycloadduct **3**. We then turned our attention to the Grubb’s catalyst, which had been used previously in [2+2+2] cycloaddition reactions by several research groups [14,15]. Treatment of **1** with a 7% molar or 20% molar of Grubb’s catalyst in refluxing toluene provided only moderate yields of **3** (from 36% to 42%). Finally, we decided to test rhodium complexes, particularly focusing on Wilkinson’s complex. In 1974, Müller [16] used stoichiometric quantities of RhCl(PPh_3_)_3_ for the cycloaddition of diynes and alkynes for the first time. We performed the cycloaddition of **1** using a 5% mol of RhCl(CO)(PPh_3_)_2_ in toluene at 65º C, affording a 96% yield of **3** (Scheme 1). Reducing the catalytic amount of Rh(I) complex to 1% molar dropped the yield of **3** to 80%. Owing to these initial results, we decided to continue our studies using rhodium complexes as the metal of choice. In the same study, we extended the cycloaddition reaction to 15-membered cyclic enediynes **4** and **5** (Scheme 2). Using either RhCl(CO)(PPh_3_)_2_ or RhCl(PPh_3_)_3_, cyclohexadiene systems **6** and **7** were obtained in excellent yields. The most important fact, however, was that the reaction proceeded with total stereoselectivity and the initial sterochemistry of the macrocyclic double bond was maintained during the cycloaddition process. In addition, no aromatization process or other side reactions took place [10] (Scheme 2).

In a collaboration project with the research group of J. Muzart at the CNRS-Université de Reims Champagne-Ardenne in France [17], we aimed to go one step further by exploring the recovery and reuse of Wilkinson’s complex in the cycloaddition of macrocycles **1** and **5**. We studied the use of molten tetra-n-butylammonium bromide as a good immobilizing agent for RhCl(PPh_3_)_3_ that allowed the catalyst to be recycled twice without a loss of activity.

**Figure 2:**
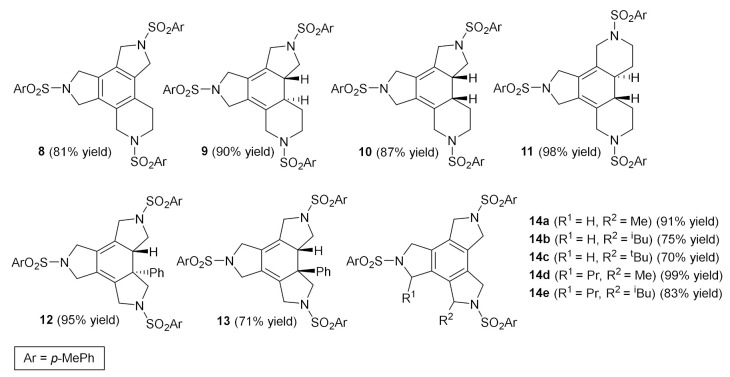
The fused tetracycles with a benzene or cyclohexadiene core obtained by the [2+2+2] cycloaddition reaction catalyzed using Wilkinson’s complex.

Encouraged by the results with our macrocyclic systems, we extended the totally intramolecular cycloaddition reaction to a large range of unsaturated azamacrocycles. The macrocycles differ in the ring size (15-, 16-, 17-, 20-, and 25-membered), the number and type of unsaturations (triacetylene and enediyne macrocycles with (Z) and (E) geometry), and the presence of substituents either in the double bond or in the *α*-position relative to the triple bond [18,19,20]. The cycloaddition reaction was run using a 10% molar of RhCl(PPh_3_)_3_ in toluene at temperatures from 60 °C to reflux affording excellent yields of the corresponding fused tetracycles with benzene and cyclohexadiene cores. Figure 2 shows the scope of the reaction.

**Scheme 3:**
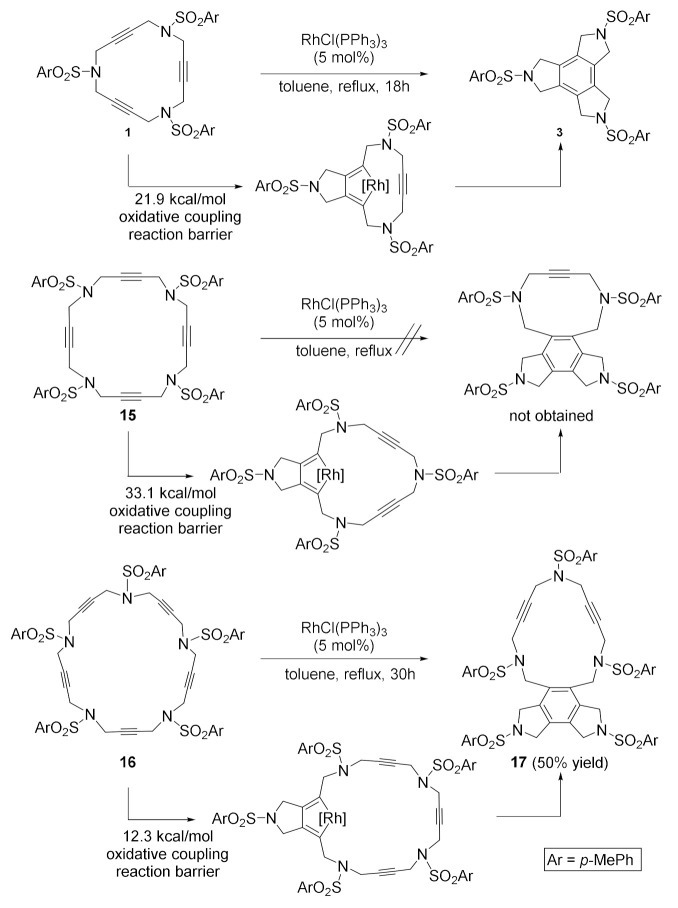
Studies on cycloaddition reactions of 15-, 20-, and 25-membered polyacetylenic azamacrocycles **1**, **15** and **16**.

The 20- and 25-membered polyacetylenic azamacrocycles need to be addressed as a separate case [20]. When we performed the [2+2+2] cycloaddition of azamacrocyles **15** and **16**, the two behaved differently (Scheme 3). The 20-membered macrocycle **15** did not lead to the expected cycloadduct compound. In contrast, when the 25-membered macrocycle **16** was treated with a catalytic amount of Wilkinson’s complex, cycloadduct **17**, resulting from the reaction of three contiguous alkynes, was obtained as the only product of the process. In order to justify the different reactivity of the 15-, 20- and 25-membered macrocycles, we performed DFT calculations supervised by Prof. Miquel Solà from the University of Girona. First, the thermodynamics for the overall Wilkinson’s complex catalyzed [2+2+2] cycloaddition was computed. For all three macrocycles, the reaction was highly exergonic: 128.4 kcal/mol for the 15-membered macrocycle **1**, 122.0 kcal/mol for the 20-membered macrocycle **15**, and 121.6 kcal/mol for the 25-membered macrocycle **16** when the reaction took place on three consecutive alkynes. Since the difference in reactivity could not be explained by the thermodynamics of the reaction, we focused on the kinetics. We evaluated the detailed reaction profile and found that the rate-determining step was the oxidative coupling and that the energy barriers computed for this step in the three macrocycles differred considerably (Scheme 3). Indeed, the barrier for the oxidative coupling in the 20-membered macrocycle **15** (33.1 kcal/mol), which experimentally failed to cycloisomerize under the reaction conditions tested, was 11.2 kcal/mol and 20.8 kcal/mol higher than those for 15-membered macrocycle **1** (21.9 kcal/mol) and 25-membered macrocycle **16** (12.3 kcal/mol), respectively. A detailed analysis showed that the deformation energy could not explain the observed differences. Two factors were proposed to accound for the absence of reactivity observed for **15**. Firstly, the main interaction in the transition state occurs between the LUMO of the catalyst and the HOMO of the macrocycle. In the 20-membered macrocycle **15**, the HOMO is more stable and delocalized than both in **1** and **16**, and thus it interacts in a less favorable manner with the LUMO of the catalyst. Secondly, a strained 10-membered ring carrying an alkyne is formed. The results obtained in the [2+2+2] cycloaddition reaction in macrocyclic systems were summarized in a review in Molecules [21].

When we started our work with these Rh-catalyzed [2+2+2] cycloaddition reactions, the mechanistic information on these processes was scarce. To the best of our knowlegde, we were the first to carry out a theoretical mechanistic study by DFT calculations of the Rh-catalyzed [2+2+2] cycloaddition to justify the contrasting behaviour of our different-sized macrocycles, vide supra. In this first study, the computational cost of the DFT studies was reduced by replacing PPh_3_ by PH_3_ in the Wilkinson’s complex. However, as we are aware that the electronic and steric effects of the PPh_3_ ligands are quite different from those created by the PH_3_ ligands, we decided to validate this ligand truncation. We thus performed a DFT study of the [2+2+2] cycloaddition of three molecules of acetylene catalyzed using Wilkinson’s complex [22]. The reaction followed the oxidative coupling/alkyne insertion/reductive elimination basic scheme (Figure 1). The energy profile derived from the real [RhCl(PPh_3_)_3_] Wilkinson’s catalyst was compared with that obtained with a model of the catalyst in which the PPh_3_ ligands were substituted by PH_3_ molecules. We concluded by DFT calculations that this substitution had little influence on the thermodynamics obtained, while the barrier of the rate-determining step was somewhat increased (about 5 kcal·mol^−1^) in the model system. These results justify the use of this simplified model of the catalyst in theoretical studies of more complex cyclotrimerizations, such as in the case of 15-, 20- and 25-membered azamacrocycles [20].

Taking into account the wealth of mechanistic information that can be obtained by combining experimental and theoretical techniques and how this information can help us to increase the efficiency of the reactions, a close collaboration with Prof. Miquel Solà has continued to this day [23].

**Scheme 4:**
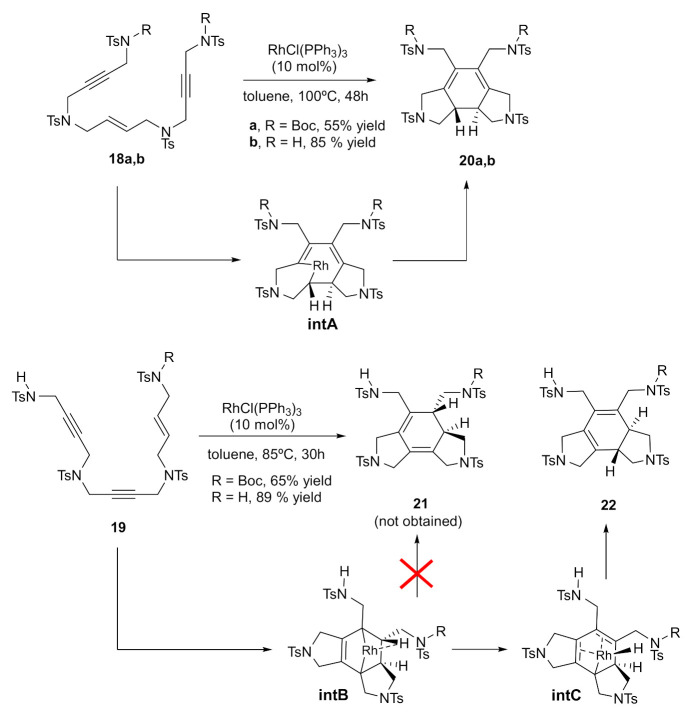
The RhCl(PPh_3_)_3_-catalyzed cycloaddition reactions of compounds **18** and **19** and the key intermediates that explain the divergent reactivity.

We then focused our attention on the Rh-catalyzed cycloaddition of a series of yne-ene-yne and yne-yne-ene open-chain enediynes (Scheme 4) [24]. Experimentally, we found that when yne-ene-yne derivatives **18a**,**b** were reacted under Rh catalysis, cycloadducts **20a**,**b**, resulting from a standard [2+2+2] cycloaddition reaction, were obtained in good yields. In contrast, when yne-yne-ene derivatives **19** were treated with catalytic amounts of Wilkinson’s catalyst, the expected derivatives **21** were not formed. Isomers **22**, with the double bonds shifted from their expected positions, were isolated in excellent yields.

DFT calculations again helped us to unravel the reasons behind the different behaviour of the two types of enediyne derivatives. The mechanistic study revealed that enediynes **19** underwent an alkyne–alkyne oxidative coupling, followed by an alkene insertion into the rhodacyclopentadiene intermediate, which formed rhodium intermediate **IntB** featuring an agostic interaction between the rhodium atom and a β-H. **IntB** did not suffer reductive elimination, but underwent a β-hydride elimination to **IntC**, followed by the reductive elimination of Wilkinson’s catalyst to yield cycloadducts **22**. In contrast, enediynes **18** evolved to rhodacycloheptadiene intermediate **IntA** that reductively eliminated rhodium to afford cycloadducts **20**.

**Figure 3:**
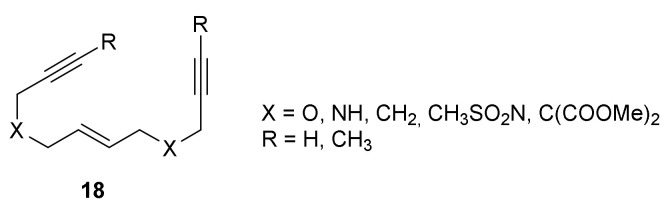
Enediynes **18** studied by DFT calculations.

After analysing the different reactivities that arise when the order of the unsaturations in the linear chain is shuffled, we decided to analyse the effect of the tether and terminal substituents on the cycloaddition of open-chain enediynes of type **18** (Figure 3) [25]. The main goal of the study was to understand the factors that had an effect on the order in which the different unsaturations entered the catalytic cycle, i.e., if the oxidative coupling involved the two alkynes or an alkyne and an alkene. The latter pathway had been proposed to lead to processes with higher enantioselectivities. The study was based on analysing the oxidative coupling step for enediynes with different tethers X (X = O, NH, CH_2_, CH_3_SO_2_N, and C(CO_2_Me)_2_) and two alkyne termini substituents (R = H and CH_3_) (Figure 3). Based on our previous studies, catalytically active species with one or two phophines coordinated to the rhodium were considered. First, we again checked the validity of using a truncated model of the catalyst. Thus, the oxidative coupling for oxygen tethered (X = O) substrates featuring either a hydrogen or a methyl attached to the alkyne (R = H and R = CH_3_) were computed for both the [RhClPPh_3_] and phosphine truncated catalyst model [RhClPH_3_]. The use of PH_3_ as a model for PPh_3_ resulted in higher computed barriers for the oxidative coupling (by 15–20 kcal/mol). However, the qualitative trends were not altered. When comparing the Gibbs energy barriers for the oxidative coupling of two alkynes (alkyne–alkyne) and an alkyne and an alkene (alkyne–alkene), both catalytic systems provided lower energy barriers for the alkyne–alkyne oxidative coupling when the substrate featured terminal alkynes (R = H) and similar barriers for the alkyne–alkyne and alkyne–alkene oxidative coupling for the substrate with an internal (R = CH_3_) alkyne. Furthermore, the two catalysts provided lower Gibbs energy barriers for terminal alkynes (R = H) as compared to internal alkynes (R = CH_3_) (ca. 10 kcal/mol and 2 kcal/mol difference in alkyne–alkyne and alkyne–alkeneoxidative coupling, respectively), in line with the experimentally observed enhanced reactivity for terminal alkynes. The work was then extended to the whole set of substrates (Figure 3) with the catalyst’s simplified model. Analogous qualitative results were obtained for X = O, NH, and CH_2_ with an effect of the tether on the oxidative coupling barrier energy, which was higher for the less electronegative tethers. On the other hand, the alkyne–alkyne coupling was favoured over the alkyne–alkene coupling both for terminal (R = H) and internal (R = CH_3_) alkynes when bulkier tethers (X = CH_3_SO_2_N and C(CO_2_Me)_2_) were present in the substrate. Finally, to discover an explanation as to why the alkyne–alkyne coupling is generally preferred, the deformation energies for the enediyne and the active catalyst were computed. The steric repulsion between the terminal groups on alkyne were found to be responsible for the higher deformation energy of enediyne in the transition state of the alkyne–alkyne coupling.

**Scheme 5:**
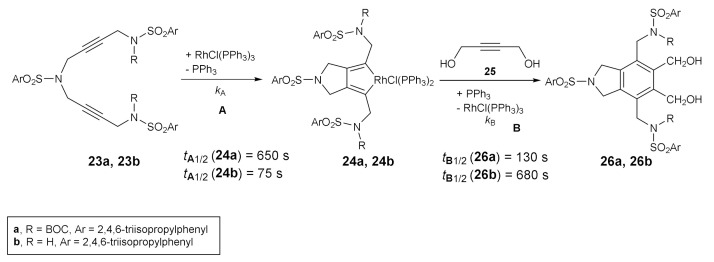
[2+2+2] Cycloadditions of diynes **23** and monoalkyne **25** studied by CV.

Another foray into the experimental mechanistic studies of these cycloadditions was made in collaboration with Prof. Anny Jutand at the Ecole Normale Supérieure in Paris [26]. In this case, kinetic data on the two main steps of the catalytic cycle for a RhCl(PPh_3_)_3_-catalyzed [2+2+2] cycloaddition were obtained by cyclic voltammetry. Diynes **23** bearing either a sterically hindered group at the sulfonamide (R = BOC), **23a**, or an H atom (R = H), **23b**, were reacted with alkyne **25** under rhodium catalysis (Scheme 5). The formation of rhodium complexes **24a** and **24b** (step A) and their further reactions with the monoalkyne **25**, which deliver RhCl(PPh_3_)_3_ and the final product (step B), were followed by means of electrochemical techniques which deliver kinetic data for the two successive separately investigated steps. The data led us to conclude that the rate-determining step differs depending on the sulfonamide substitution. The formation of the rhodacyclopentadiene (step A) is the rate-determining step for **23a** (R = BOC), whereas for the less-bulky diyne **23b** (R = H), the insertion of the 2-butyn-1,4-diol to the rhodacyclopentadiene and subsequent recovery of the catalyst (step B) determines the rate of the overall reaction. The results of these kinetic studies indicate that the first or second step in the cycloaddition may be rate-determining, depending on the structure of the starting reagents.

**Scheme 6:**
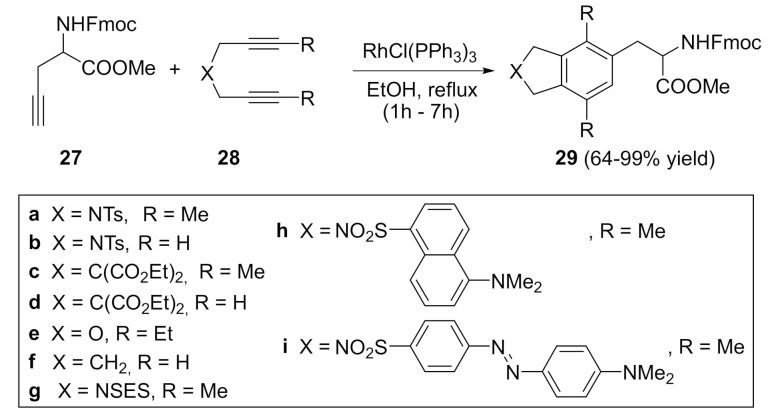
The Rh-catalyzed [2+2+2] cycloadditions of L,D-**27** and L-**27** with diynes **28**.

Since we had demonstrated the high activity of Wilkinson’s catalyst in our macrocyclic compounds, open-chain enediyne substrates, and in the cycloaddition of diynes and alkynes, we decided to go further and study the application of these cycloadditions to the synthesis of high-added-value compounds such as non-proteinogenic aminoacids [27] (Scheme 6). We prepared a wide range of phenylalanine derivatives of type **29** by the cycloaddition of several stereochemically pure and racemic protected propargylglycine derivatives L-**27** and L,D-**27** with diynes **28,** featuring different tethers. Curiously, the cycloaddition using the RhCl(PPh_3_)_3_ complex was highly dependent on the solvent that was used in the reaction. When toluene or CH_2_Cl_2_ were used, as in our previous cases, the reaction failed. However, using EtOH as the solvent at refluxing temperature, the cycloaddition worked efficiently, affording a wide range of aminoacid derivatives, **29a**–**29i**, with excellent yields. With the different diynes used, it was possible to introduce diverse functionalities in the amino acids **29**. Fluorescent tags such as dansyl and dabsyl could be introduced into the final aminoacid, producing fluorescent-labelled biomolecules (**29h** and **29i**). The reaction conditions used were mild enough to be compatible with the acid- and base-labile protecting groups normally required for peptide synthesis and to avoid the racemization of the stereogenic centres of the starting material (Scheme 6). 

**Scheme 7:**
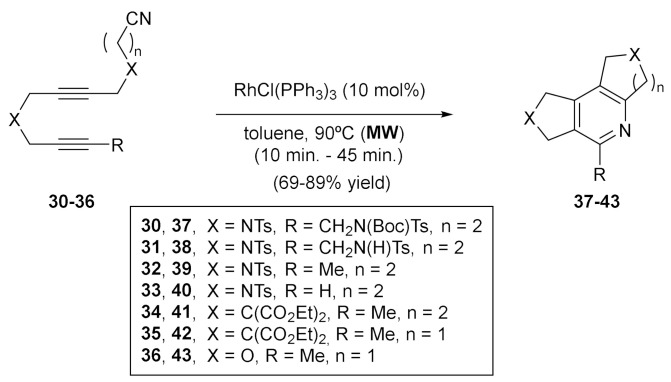
The Rh-catalyzed [2+2+2] cycloadditions of cyanodiynes **30**–**36**.

**Scheme 8:**
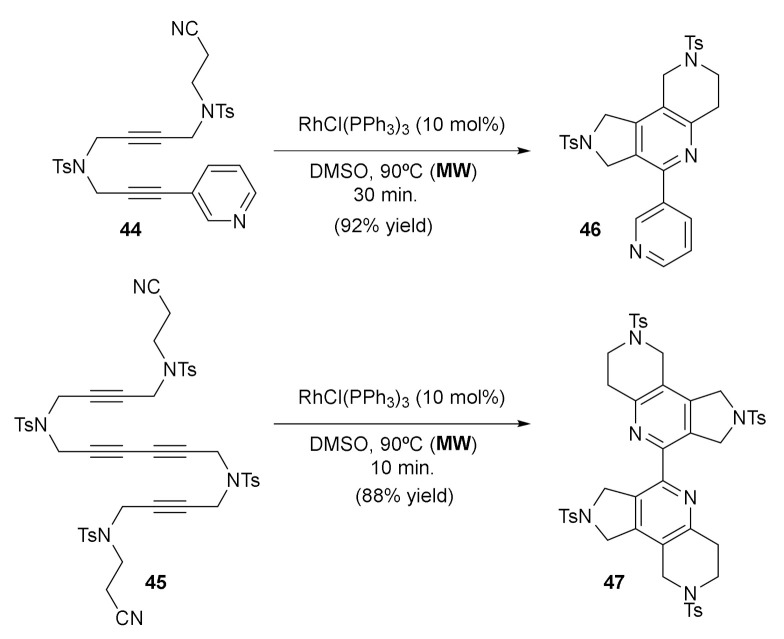
The Rh-catalyzed [2+2+2] cycloadditions of cyanodiyne **44** and dicyanotetrayne **45**.

After all these studies, we wanted to explore other unsaturations apart from alkynes and alkenes. We first centered our attention on nitriles, since the Co and Ru-catalyzed [2+2+2] cycloaddition of two alkynes and one nitrile is an efficient and straighfordward way to construct pyridine derivatives [28,29]. Examples of the use of rhodium in such processes were scarce; thus, we wanted to explore it further. We studied the completely intramolecular cycloadditions of cyanodiynes to afford highly functionalized tricyclic-fused pyridines and bipyridines catalyzed with Wilkinson’s complex [30] (Scheme 7). The cycloaddition of N-tosyl tethered cyanodiynes **30**–**33** using a 10% molar of RhCl(PPh_3_)_3_ in refluxing toluene required long reaction times and considerable amounts of decomposition products were formed, reducing the yield of the corresponding pyridine derivatives **37**–**40**. In order to minimize the decomposition processes, the cycloaddition was carried out under microwave irradiation and, as expected, the reaction time was considerably reduced and the yields of **37**–**40** were significantly increased. The methodology under microwave heating was extended to malonate-tethered cyanodiynes **34**–**35**, oxygen-tethered cyanodiyne **36** (Scheme 7), and cyanodiyne **44** and dicyanotetrayne **45** (Scheme 8). In the case of derivatives **44** and **45**, cycloaddition led to the formation of bipyridines **46** and **47**, and very good yields were obtained when the reaction solvent was changed from toluene to DMSO, a higher microwave-absorbing solvent (Scheme 8).

In addition, the mechanism of the RhCl(PPh_3_)_3_ catalyzed [2+2+2] cycloaddition between two acetylene molecules and several nitriles for the formation of pyridines was computationally studied [31]. The oxidative coupling of the two alkynes was more favourable than the oxidative coupling between an alkyne and the nitrile moiety. However, the insertion of a third alkyne molecule to the rhodacyclopentadiene was somewhat more favourable than the insertion of the nitrile, providing an explanation for why an excess of the nitrile derivatives is usually required in these cycloadditions.

After demonstrating the high level of activity and the versatility of Wilkinson’s complex as a catalyst in the field of [2+2+2] cycloadditions involving nitriles, we focused on involving allenes as unsaturated substrates. Allenes are cumulated systems with two contiguous carbon–carbon double bonds. They are challenging substrates in cycloaddition reactions because the selectivity control is more complex than in the case of the use of alkynes or alkenes. Apart from the chemoselectivity towards other unsaturations and the issues of regio- and stereoselectivity, the chemoselectivity as to which of the two double bonds will react also needs to be controlled.

**Scheme 9:**
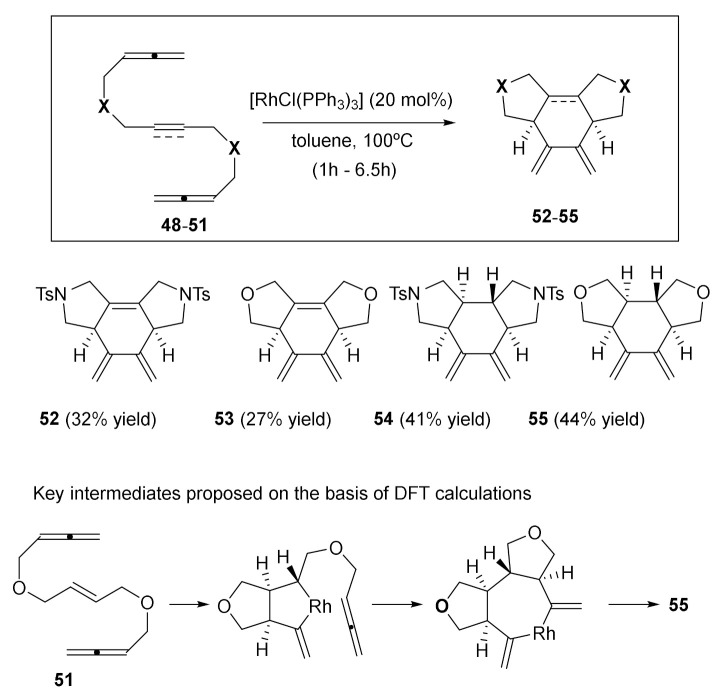
The [2+2+2] cycloadditions of the allene–yne–allene derivatives **48*–*49** and the allene–ene–allene derivatives **50*–*51** catalyzed by the RhCl(PPh_3_)_3_ catalyst.

The first substrates that we prepared for the evaluation of their [2+2+2] cycloaddition were linear allene-ene/yne-allene derivatives **48**–**51** [32] (Scheme 9). We again tested the activity of Wilkinson’s catalyst in these processes and, as we envisaged, the tricyclic cycloadducts **52**–**55** were obtained in moderate yields. The cycloaddition was completely chemo- and diastereoselective. All four cycloadducts were formed by a chemoselective reaction of the internal double bond of the two allenes. In addition, the most remarkable fact was that substrates bearing a double bond in the central position (**50** and **51**) yielded a tricyclic cycloadduct in a reaction in which four contiguous stereogenic centres were formed as a single diastereoisomer.

The diastereoselectivity and the preference for cyclisation at the internal double bond of the allene were analyzed using DFT calculations (Scheme 9). The mechanistic study revealed that oxidative coupling takes place between the central alkene and the internal double bond of one of the allenes, leading to a cis-fused bicyclic intermediate. The second allene is then inserted into the rhodium-Csp^3^ bond of the rhodacyclopentane to afford a trans-fused ring junction, intermediate, that then evolves to the final product **55** as the only diastereoisomer (Scheme 9).

**Figure 4:**
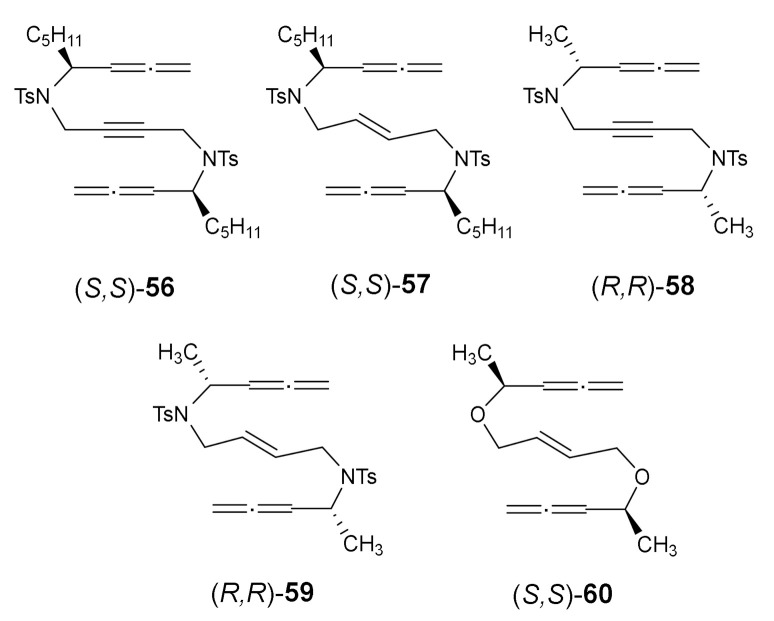
Stereopure allene–yne/ene–allene derivatives **56*–*60**.

To access the chiral polycycles from allene-yne/ene-allene derivatives, we decided to test a central-to-central chirality induction process in a collaboration project with Prof. Jordi Garcia from the Department of Chemistry at the University of Barcelona [33]. We wanted to control the configuration of the two or four stereogenic centres generated in the cycloaddition by the influence of two chiral centres that were already present in the substrate. To this end, five model compounds bearing N-tosyl- (NTs) and oxygen (O) linkage were prepared. Two stereogenic centres were situated in the α-position of the allene functionality, a long alkyl chain C_5_H_11_ and a methyl group, **56**–**60**. The substrates had either a double or triple bond in the centre of the chain (Figure 4).

**Scheme 10:**
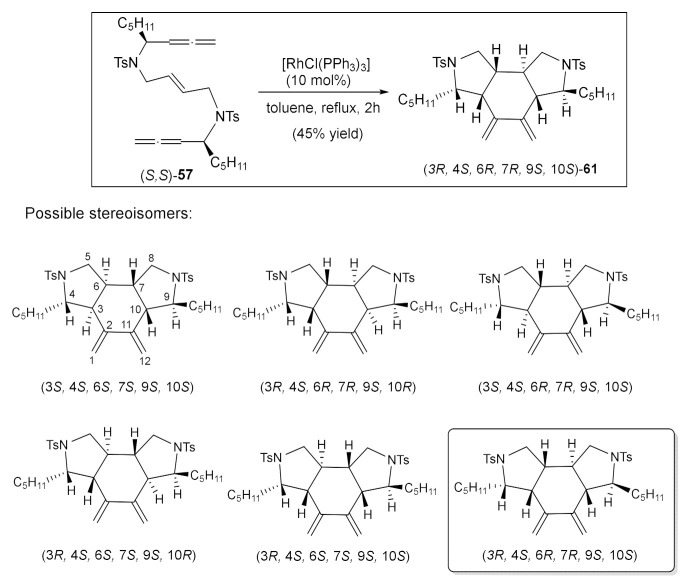
The RhCl(PPh_3_)_3_-catalyzed [2+2+2] cycloaddition of allene–ene–allene (S,S)-**57** and possible stereoisomers that could be formed upon cycloaddition.

We will comment here on only one particularly illustrative case: the cycloaddition of derivative (S,S)-**57**. The cycloaddition of **57** took place using a 10% molar of RhCl(PPh_3_)_3_ at refluxing toluene, affording a single diastereisomer of cycloadduct **61** with a 45% yield (Scheme 10). In the case of bisallene **57**, six possible stereoisomers can be formed upon [2+2+2] cycloaddition. A complete NMR study allowed us to identify the stereoisomer obtained as optically pure enantioisomer (3R,4S,6R,7R,9S,10S)-**61**, demonstrating that a completely diastereoselective process had taken place. The chirality of the starting bisallene substrate can be completely transferred to the cycloadduct, representing an atom-economical and enantiospecific process for the construction of fused funcionalized polycycles (Scheme 10). This study prompted us to write a review article based on the diastereoselective [2+2+2] cycloaddition reactions of enantiomerically pure substrates considering two different approaches: chirality induction and chirality transfer [34].

**Scheme 11:**
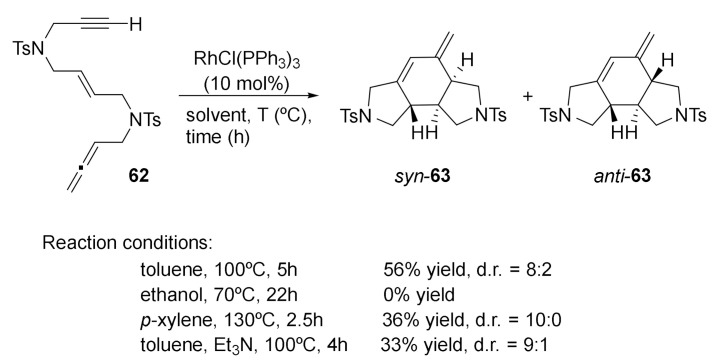
Allene–ene–yne **62** treated with RhCl(PPh_3_)_3_ at different solvents and temperatures.

Due to the versatility we found in the use of rhodium-catalyzed cycloaddition reactions involving allenes, we were interested in studying [2+2+2] cycloaddition reactions using substrates with three different unsaturations: an allene, an alkene, and an alkyne. We wanted to compare the reactivity of the three different unsaturations from both experimental and theoretical points of view [35]. To this end, allene–ene–yne substrate **62** was prepared in which the oxidative coupling of the alkyne and the allene is not geometrically favoured (Scheme 11). We started our study using a 10% molar of Wilkinson’s catalyst in refluxing toluene. A tricyclic derivative was obtained as a mixture of two diastereoisomers: syn-**63** and anti-**63**. The reaction was completely chemoselective, taking place only at the internal double bond of the allene (analogous to what we previously observed with substrates **48**–**51**). However, two diastereoisomers were formed, which differed in the 6:5 ring fusion that was either syn or anti. When different reaction conditions were tested, changing the solvent and the reaction temperature (Scheme 11), the syn isomer was remained the major isomer.

As we noted in the introduction, the fine-tuning of metal/ligand combinations and the reaction conditions can change, among other aspects, the diastereoselectivity of the cycloaddition. Therefore, we decided to try alternative rhodium-based catalytic systems in order to see if it was possible to change the diastereoselectivity of the reaction. The results obtained with alternative Rh-complexes will be explained in the following sections, along with the mechanistic study using DFT calculations that helped us to rationalize the different diastereoselectivities obtained using different rhodium complexes. 

Our contribution to the [2+2+2] cycloaddition reactions involving allenes prompted us to write a tutorial review in Chemical Society Reviews with the aim of proving that allenes are versatile unsaturated motifs in transition-metal-catalysed [2+2+2] cycloaddition reactions [36].

**Scheme 12:**
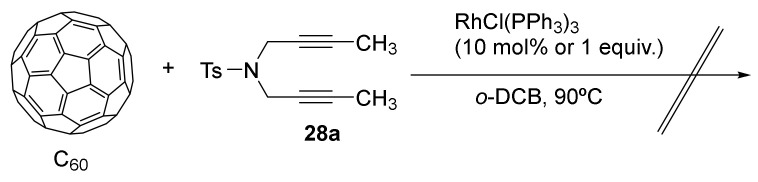
The failed attempts at the RhCl(PPh_3_)_3_-catalyzed [2+2+2] cycloaddition of C_60_ with diyne **28a**.

During the last five years, we have focused our methodological studies on the application of Rh-catalyzed [2+2+2] cycloaddition reactions in material science, specifically in the field of photovoltaic devices. Sunlight can be harvested by means of solar cells, devices that convert the absorbed incident sunlight into electricity. Perovskite solar cells (PSCs) are one of the most promising candidates for solar cells due to their outstanding photovoltaic performance and simple and low-cost manufacturing process. Of particular interest are certain small molecules, such as fullerene derivatives, that have been explored as additives or surface modifiers in PSCs, since they successfully passivate the interface trap states at the grain boundaries [37]. Chemical functionalization of the fullerene cage offers many ways to tune their photophysical and electrochemical properties to a specific application. Therefore, we wanted to explore the [2+2+2] cycloaddition of alkynes and C_60_—considered as an electron poor alkene—as a means to functionalize the fullerene cage with different addends. We started by using DFT calculations to evaluate whether the cycloaddition of C_60_ with two alkynes in the presence of Wilkinson’s catalyst was energetically feasible [38]. The mechanistic study demonstrated that Wilkinson’s complex was able to catalyze the [2+2+2] cycloaddition of C_60_ and two acetylene molecules to form a cyclohexadiene ring fused to a [6,6] bond of C_60_. The energetically more favourable reaction pathway involved an oxidative coupling of the two acetylene molecules with rhodium, followed by the insertion of C_60_ into a Rh–C bond of the rhodacyclopentadiene intermediate formed. A deeper analysis of the mechanism indicated that the use of an excess of C_60_ was likely necessary to avoid side reactions such as the formation of benzene and that the energy barriers could be reduced by using diynes instead of acetylene. We thus began the experimental study by reacting C_60_ with diyne **28a** using Wilkinson’s catalyst. However, this catalyst did not promote the reaction, even when stoichiometric amounts of catalyst were used [39] (Scheme 12). Susprised by the failure of the Wilkinson’s complex, we moved on to other rhodium-based catalytic systems, as will be explained in the following section.

### 2.2. Combinations of Rh Complexes with Phosphines

Wilkinson’s catalyst was the first complex introduced in homogeneous catalysis for the hydrogenation of olefins and has found vast application to other homogeneous catalysed transformations including the [2+2+2] cycloaddition, as outlined above. However, regarding chirally assisted processes, the use of combinations of rhodium sources with atropoisomeric biphosphines, either in the form of a preformed catalyst or generating in situ chiral rhodium complexes, provide a very powerful and flexible way to improve catalytic activity.

**Scheme 13:**
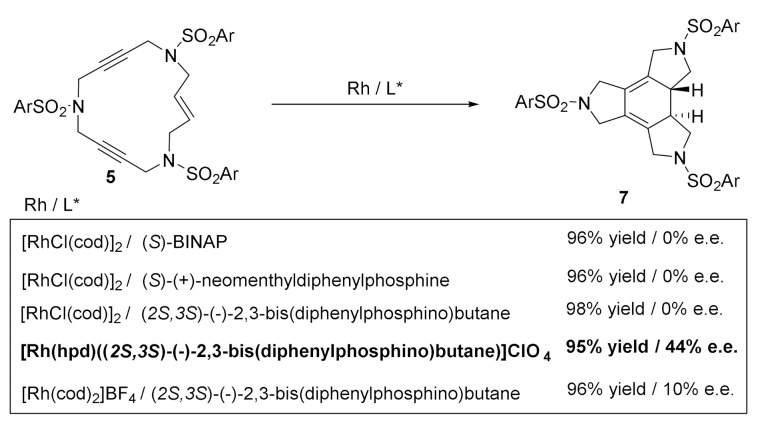
The enantioselective [2+2+2] cycloadditions of enediyne azamacrocycle **5**. L* = chiral ligand.

We first tested the use of combinations of Rh complexes with phosphines on the [2+2+2] cycloaddition of enediyne macrocycles (Scheme 13) [18]. The enantioselective cycloaddition of **5** would form enantioenriched cyclohehexadienes with two stereogenic carbon centers. When we explored the enantioselective cycloaddition of macrocycle **5**, none of the reactions using [RhCl(cod)]_2_ and chiral phosphines with different steric hindrances led to chirality induction, but the use of the cationic catalyst Rh(hpd)((2S,3S)-(−)-2,3-bis(diphenylphosphino)butane) (hpd = bicyclo[2.2.1]hepta-2,5-diene) with the chiral ligand already coordinated (i.e., not formed in situ) induced up to a 44% of enantiomeric excess (e.e.), with an excellent yield of 95% of **7**.

**Scheme 14:**
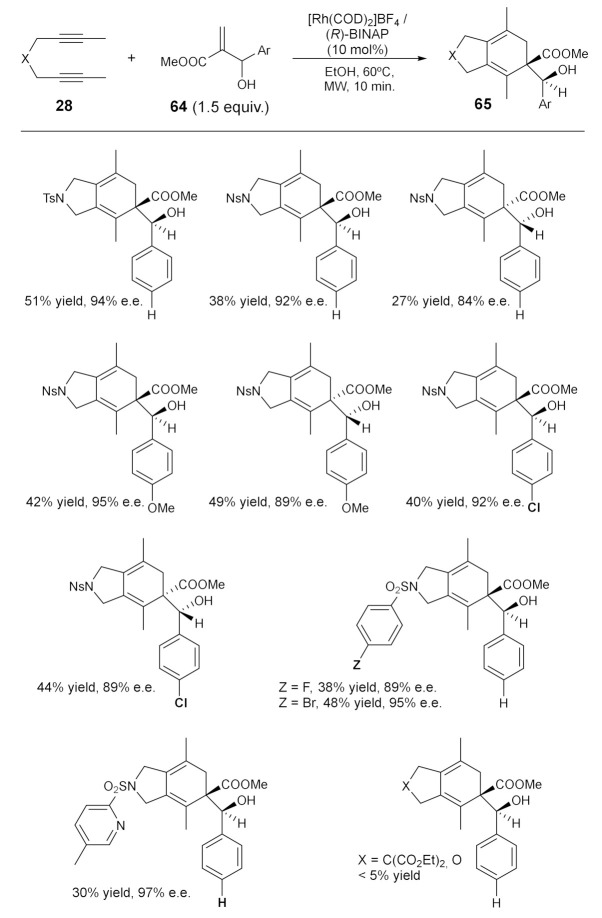
The rhodium-catalyzed [2+2+2] cycloadditions of diynes **28** with MBH adducts **64**.

Considering the potential of alkenes as unsaturations in these processes, one of our contributions in this field, again in collaboration with the Muzart’s group in Reims, has been the use of Morita–Baylis–Hillman (MBH) adducts **64** as alkenes (Scheme 14) [40]. These derivatives are densely functionalized molecules, containing at least three functional groups in proximity. The cycloaddition of the adducts with diynes **28** may be a means to the enantioselective synthesis of cyclohexadiene derivatives containing vicinal tertiary and quaternary carbon centres. With this transformation in mind, we hypothesized that the presence of an alcohol next to the double bond would enhance stereodiscrimination through substrate chelation (Scheme 14).

We set up the reaction conditions with an N-tosyl-tethered diyne and, after some experimentation, the cycloaddition took place using a combination of a cationic rhodium complex and racemic-BINAP as the ligand. NMR analysis of the cycloadduct showed the formation of only one of the two possible diastereoisomers that can be formed. We then studied the stereoselectivity of this cycloaddition. After testing several chiral biphosphines, the use of (R)-BINAP in EtOH under microwave heating led to a 51% yield of the product, with excellent enantioselectivity. Having started from a racemic MBH adduct, an enantiopure cycloadduct was obtained, suggesting that a kinetic resolution process was operative. In addition, the use of (S)-BINAP promoted the reaction of the other enantioisomer of the B-H adduct and afforded the (S,R)-product with an 81% e.e.. Scheme 14 shows the scope of the process. The results showed that the tether in the diyne was crucial for the efficiency of the process, with good results obtained only for sulfonamide tethers. The reaction tolerated every substitution in the aromatic ring of the sulfonamide, including a pyridine ring whose nitrogen atom did not seem to poison the catalyst.

**Scheme 15:**
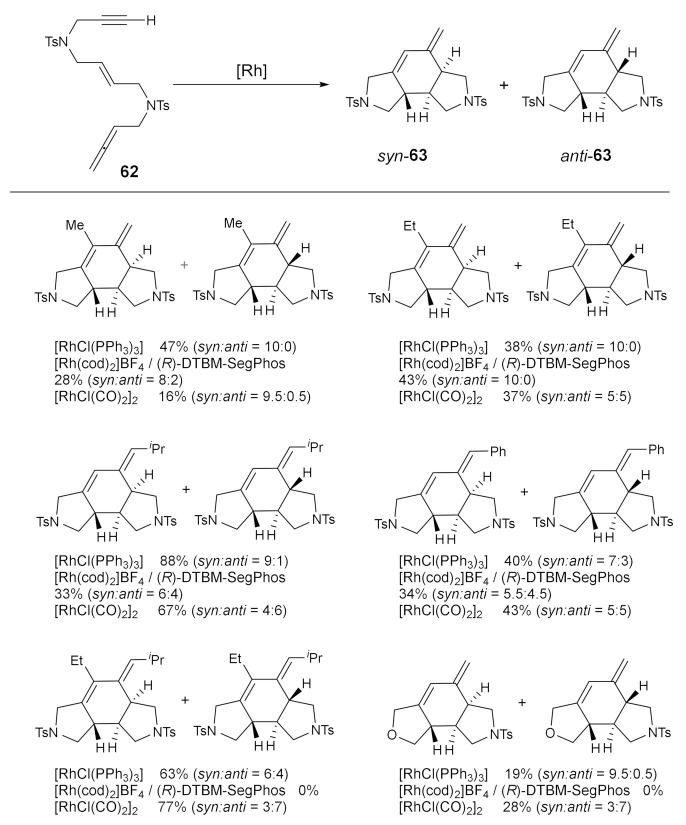
The scope of the rhodium-catalyzed [2+2+2] cycloaddition of open-chain yne–ene–allene derivatives.

As we have commented, one of the cases we studied involving allenes was the cycloaddition of linear substrates containing three different unsaturations: an allene, an internal alkene, and an alkyne [35] (compound **62** in Scheme 11). As shown in Scheme 11, the feasibility of the cycloaddition was assessed using Wilkinson’s catalyst in toluene at 100 °C. The reaction was completely chemoselective, with cycloaddition taking place only in the internal double bond of the allene. However, two diastereisomers were obtained that differ in the 6:5 ring fusions that can be either syn or anti. We then decided to test several other rhodium catalytic systems in order to improve the selectivity of the process. We found that changing phosphine to carbonyl ligands on rhodium altered the selectivity of the process. The use of Segphos with cationic rhodium complex [Rh(cod)_2_]BF_4_ did not improve the yield or the ratio. The scope of the process is detailed in Scheme 15. In general terms, we found that the Wilkinson’s catalyst displayed a higher selectivity for the syn cycloadduct. When methyl and ethyl substituents were placed at the terminus of the alkyne group, pure syn diastereoisomers were obtained in moderate yields. However, the stereoselectivity was reduced when the steric hindrance of the groups at the ends of the unsaturations was increased. On the other hand, the rhodium carbonyl complex [RhCl(CO)_2_]_2_ was only moderately selective towards the anti cycloadduct.

The results of this study allowed us to compare three different rhodium catalysts, all of which were able to promote the cycloaddition chemoselectively involving the internal double bond of the allene. However, the diastereoselectivity of the reaction proved to be not only catalyst dependent, but also substrate dependent. Again, DFT calculations of the mechanism of this cycloaddition helped us to understand the differences between the two best catalytic systems. Comparison of the two calculated catalytic cycles using [RhCl(PPh_3_)_3_] and [RhCl(CO)_2_]_2_ allowed us to computationally explain the experimental detection of two diastereoisomers since very low energetic differences were found between similar potential energy surfaces leading to the syn and the anti diastereoisomers. Furthermore, the difference in selectivity of these two catalytic systems can be attributed to the variation in the coordination sphere of the catalytically active species: the [RhCl(PPh_3_)_3_] catalyst cycle runs using a catalytic species with only a chloride ligand, while the outcome for the [RhCl(CO)_2_]_2_ catalyst relies on the number of carbonyl units coordinated to the metallic centre [35]. With this study, we have confirmed that fine-tuning the rhodium/ligand combination can provide different results in terms of yields and diastereoselectivity.

**Scheme 16:**
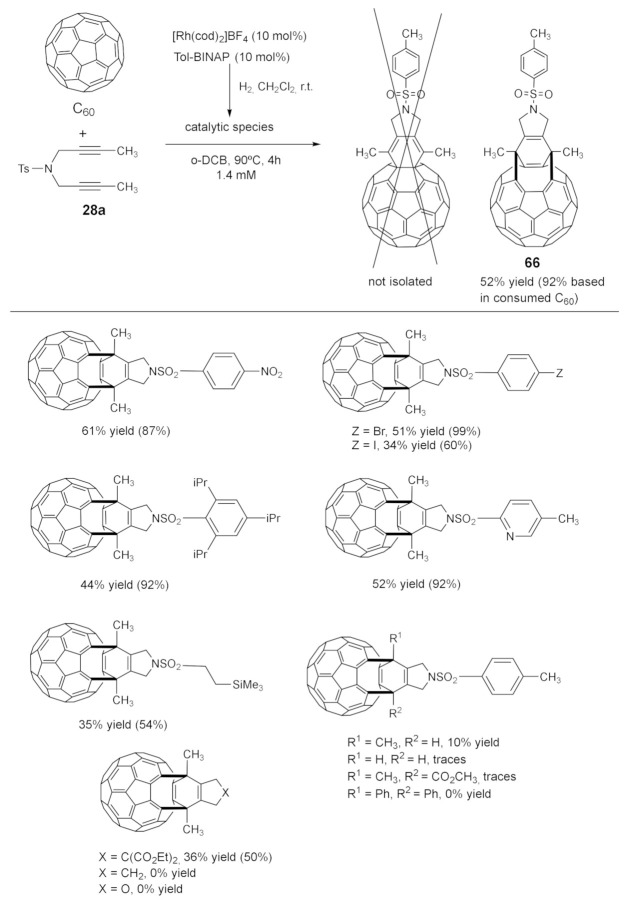
The rhodium-catalyzed [2+2+2] cycloadditions of diynes with C_60_.

In the case of involving fullerenes in the cycloaddition with diynes (see Scheme 12), we shifted to the use of [Rh(cod)_2_]BF_4_ and several biphosphines, since Wilkinson’s catalyst failed in this cycloaddition [39] (Scheme 16).

We tested different biphosphines (BINAP, H_8_-BINAP, BIPHEP, DTBM-Segphos, DPPE, DPPF, and Tol-BINAP), solvents, and temperatures; the best reaction conditions were obtained using 10% mol of [Rh(cod)_2_]BF_4_ and Tol-BINAP in o-dichlorobenzene at 90 °C over 4 h. After a complete NMR study, we realized that the expected cyclohexadiene was not isolated, but rather that the corresponding open-cage fullerene **66** with a C_s_ symmetry was formed. We were very pleased with this result, as we had established a straightforward entry to open-cage fullerenes based on a cascade process triggered by the Rh-catalyzed [2+2+2] cycloaddition and followed by fullerene-cage opening. The well-established synthetic entry to open-cage fullerenes from cyclohexadiene-fused C_60_ is the photoinduced ring-opening, which was not the case in our study. Given that open-cage fullerenes can play an important role as molecular containers, we decided to study the scope of the process (Scheme 16). Different substituents in the sulfonamide were tolerated, as well as iodo and bromo benzene derivatives, which have the potential for further functionalization. Non-aromatic sulfonamides and other tethers, such as malonates, were also involved in the cycloadditions. The O-tether seems to be more critical, and no reaction took place.

**Scheme 17:**
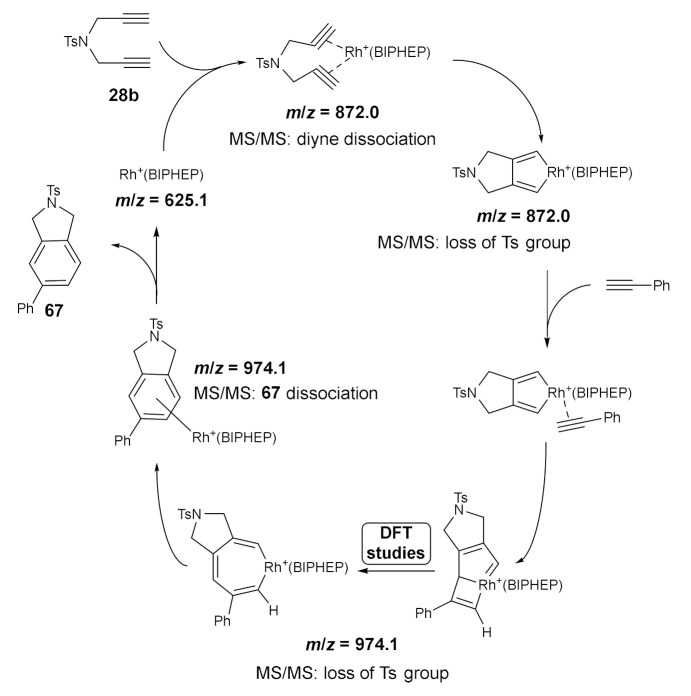
The catalytic cycle proposed for the Rh^+^/BIPHEP-catalyzed [2+2+2] cycloaddition of diyne **28b** and monoalkyne with detected ESI-MS species.

An experimental-theoretical mechanistic study using a [Rh(cod)_2_]BF_4_/biphosphine catalytic system was carried out with the aim of characterizing relevant intermediates in the cycloaddition reaction between diyne **28b** and phenylacetylene using electrospray ionization mass spectrometry (ESI-MS) [3]. This technique is particularly useful for detecting and trapping short-lived and thermally sensitive intermediates, especially in transition metal catalyzed processes. One of the characteristics of this soft ionization mass technique is the direct detection of short-lived intermediates throughout the on-going reaction [41]. The cationic nature of the reaction intermediates provided by the inherently cationic catalytic system, as opposed to the neutrality of the reactants and the products of the reaction, was key to the observation of cycloaddition intermediates in low concentrations, which other techniques have not been able to characterize. In Scheme 17, species detected by ESI-MS, as well as the proposed catalytic cycle, are shown. All the species detected were further characterized by MS/MS analysis. The species with rhodium attached covalently to the substrates suffered the loss of tosyl groups by a homolytic cleavage, affording cationic radical fragments. These ESI-MS/MS experiments helped us to identify addition and insertion intermediates through the synthetic pathway.

However, ESI-MS failed to provide structural information for the second intermediate. All the different possibilities (**V**, **VI** or **VII**, Figure 1) have the same mass and the observed fragmentation is not characteristic of a specific structure. We thus decided to perform DFT calculations. The mechanism for the alkyne insertion was computed, and two different intermediates were localized in the potential energy surface. Alkyne insertion initially led to rhodabicyclo[3.2.0]heptatriene that evolved though a Gibbs energy barrier of 1.7 kcal/mol to rhodacycloheptatriene in a process exergonic by 11.8 kcal/mol. From the latter, a reductive elimination surpassing a barrier of 2.2 kcal/mol delivered the final product. Thus, the species observed by ESI-MS at a m/z = 974.1 likely correspond to the most stable rhodacyclopentadiene, although the small energy difference between the two intermediates prevents a firm conclusion.

This study was one of the first experimental studies that allowed the direct detection of the elusive intermediates resulting from the insertion of the phenylacetylene into the rhodacyclopentadiene intermediate.

Another experimental-theoretical mechanistic study that our group perfomed was based on the analysis of the [2+2+2] cycloaddition of several p-X-substituted phenylacetylenes catalyzed by [Rh(BIPHEP)^+^ [42] (Table 1). Since the regioselectivity is an important aspect when the cycloaddition take place within three identical monosubstituted alkynes, the aim of this study was to analyze the effect of the electronic character of the phenyl substituents on the regioselectivity. We started by computing the whole mechanism using DFT calculations on three p-X-substituted phenylacetylenes with different electronic demands (X = H, NO_2_ and NH_2_). Both the reaction path that has the lowest energy barrier and the one with the most stable transition state (Curtin–Hammett principle) were considered for each substrate, and product ratios were predicted based on rate constants from the transition state theory and the Gibbs energy barriers of the pathways that led to the different regiosiomers. Oxidative coupling of p-nitrophenylacetylene, which bears the most electron-withdrawing substituent, selectively delivers 1,4-di-p-nitrophenylrhodacyclopentadiene. This is formed through the lowest energy barrier and has the highest stability among all the possible oxidative coupling intermediates. Alkyne insertion on this intermediate exclusively generates the 1,2,4-regioisomer, allowing us to predict a 100:0 (1,2,4-:1,3,5-) ratio. For phenylacetylene and p-aminophenylacetylene 1,3-disubstituted rhodacyclopentadiene, intermediates were computed to be preferentially formed, eventually evolving into a mixture of 1,2,4- and 1,3,5- regioisomers. The ratios of 1,2,4-:1,3,5- products computed were 99:1 for phenylacetylene and 54:46 for the p-aminophenylacetylene. We then proceeded to carry out the reactions experimentally, confirming in a very accurate manner the ratios computationally obtained (100:0 for X = NO_2_, 96:4 for X = H, and 59:41 for X = NH_2_ in the computational study and X=NMe_2_ in the experimental one). Finally, the reaction was experimentally extended to a series of phenylacetylenes with X = H, NO_2_, NMe_2_, F, Me, ^t^Bu, and OMe and good correlation was observed, both experimentally and computationally, between the regioisomeric ratios between the Hammett σ-para constant (accounting for the electronic nature of the substituents) and the regioisomeric ratios obtained experimentally.

### 2.3. Combinations of Rh Complexes with Chiral N-Phosphino Sulfinamide and Iminophosphorane Ligands

**Scheme 18:**
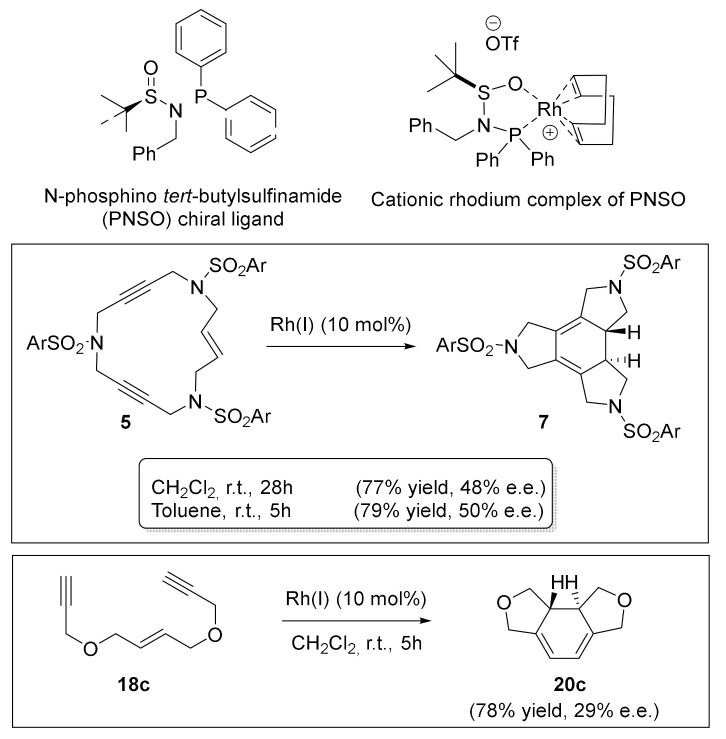
The enantioselective [2+2+2] cycloaddition of enediyne macrocycle **5** and enediyne **18c**.

Interested in the enantioselective version of these [2+2+2] cycloaddition reactions, we established a collaboration with Prof. A. Riera from the Institute for Research in Biomedicine (IRB) at the University of Barcelona. Since we had preliminary results in the enantioselective cycloaddition of macrocyclic enediyne **5** using chiral biphosphines type ligands (see Scheme 13), we moved on to other types of chiral ligands. Riera’s research group prepared a new class of chiral bidentate ligands: N-phosphino tert-butylsulfinamides [43]. These ligands efficiently combine the easily accessible sulfur chirality with the coordinating capacity of phosphorous. In addition, they have proved to be highly efficient in the intermolecular asymmetric Pauson–Khand reaction with cobalt [44]. The PNSO-Rh chiral complex proved to be effective in the cycloaddition of the enediyne macrocycle **5** (Scheme 18), but only moderate enantiomeric excesses of the cycloadduct **7** were obtained. We also tested an open-chain O-tethered enediyne **18c**. Cycloadduct **20c** was obtained with a 78% yield, but with an e.e. of only 29%. We attributed the high activity of these complexes to the hemilabile nature of the PNSO ligands. These ligands can provide vacant coordination sites to the substrate that accelerate the cycloaddition process, but, on the other hand, the chiral information is located at the hemilabile moiety of the ligand, which can be located far from the metal centre in the crucial C–C bond forming step.

**Scheme 19:**
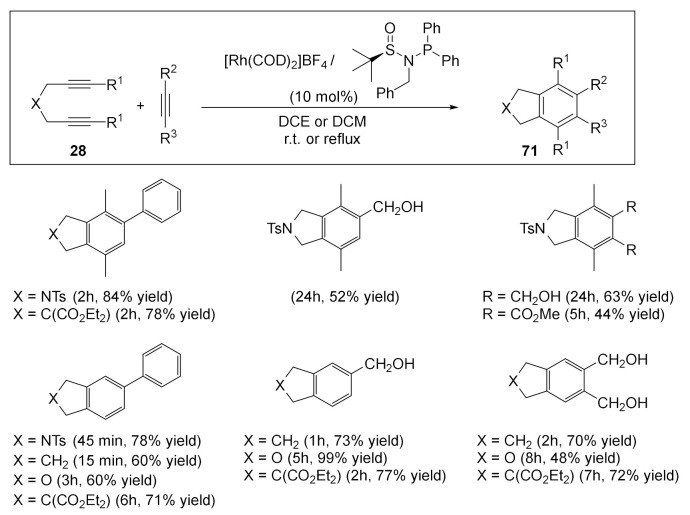
The Rh(I)-PNSO catalyzed [2+2+2] cycloaddition of diynes **28** and alkynes.

However, since the [2+2+2] cycloaddition has been accelerated by the PNSO ligands, we decided to test them in the intermolecular [2+2+2] cycloaddition reactions of diynes **28** and monoalkynes (Scheme 19). For a wide range of terminal and non-terminal diynes with different tethers (tosyl, malonates, oxygen, methylene-tethered) and with mono- and disubstituted alkynes, excellent yields of the corresponding cycloadducts **71**, in mild reaction conditions and with short reaction times, were obtained.

**Figure 5:**
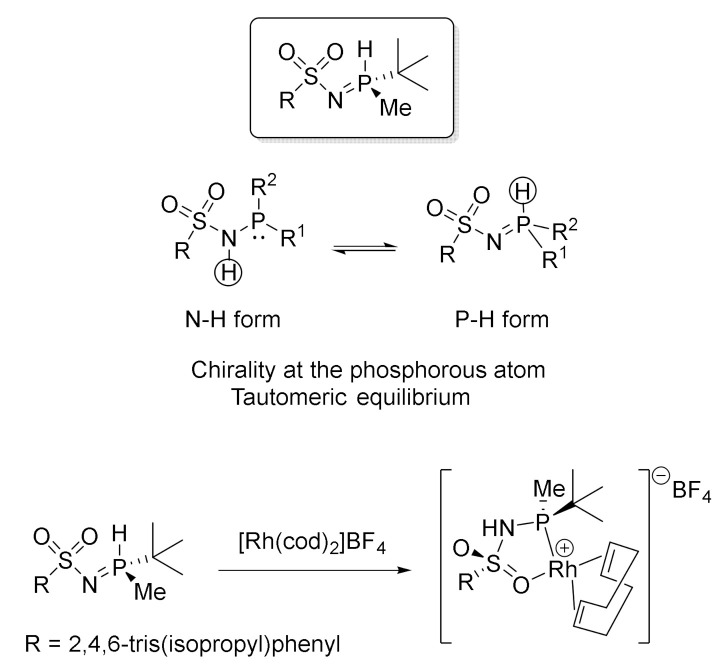
The structure of the secondary iminophosphoranes and its Rh(I) complexes.

Two years later, with the aim of improving the enantioselectivity in the cycloaddition of our enediyne systems, we tested a second type of chiral ligands designed by the same group in Barcelona [45]: P-stereogenic secondary iminophosphorane ligands (SIP). Secondary iminophosphoranes posses chirality at the phosphorous atom, and they present a PH/NH tautomeric equilibrium that preserves the chiral information. Moreover, the tautomerism renders these ligands stable to oxidation, since the secondary iminophosphorane form is almost exclusively the tautomer found in solution (Figure 5). Therefore, we decided to test the second family of Riera’s ligands.

The SIP-Rh complex (Figure 5) was isolated as an air-stable, yellow, solid monomeric species. Upon coordination to rhodium, the tautomeric equilibrium is effectively displaced towards the phosphinosulfonamide form. In these complexes, the chirality remains in the tert-butylmethylphosphino moiety, which is strongly attached to the rhodium centre, in contrast to the hemilabile sulfonyl group.

**Scheme 20:**
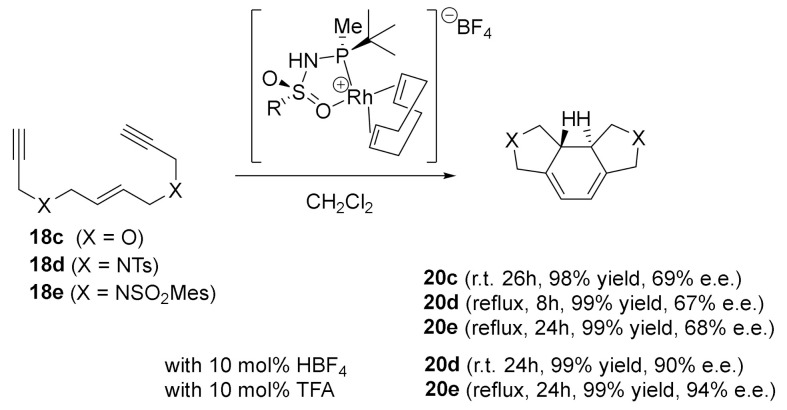
[2+2+2] The intramolecular cycloaddition of enediynes **18c**–**e** catalyzed by the SIP-Rh complex.

When we tested the SIP-Rh(I) complex for the enantioselective [2+2+2] cycloaddition of enediynes **18c**–**e**, cycloadducts **20c**–**e** were obtained in quantitative yields and e.e. was around 67–69% (Scheme 20). After a process of optimization, the presence of an acid as an additive permitted an increase of the e.e. from 67% to an excellent 94%. Most remarkably, the selectivities found in this study were, at the time, the highest that had been reported for the cycloaddition of these substrates.

### 2.4. Combinations of Rh Complexes with Dendritic Phosphoramidite Ligands

**Scheme 21:**
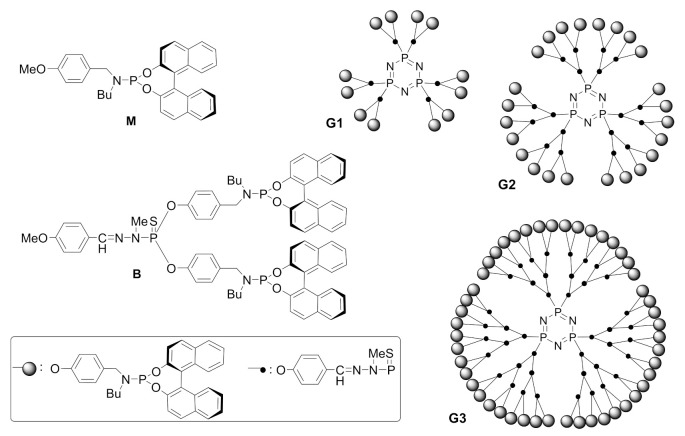
Dendrimers **G1**, **G2** and **G3**, monomer **M**, and branch **B** models synthesized.

**Scheme 22:**
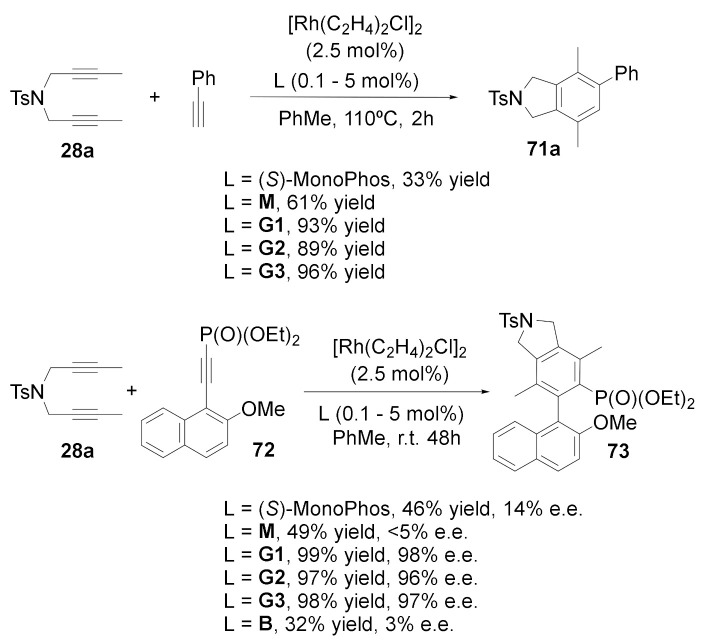
[2+2+2] The cycloaddition of three alkynes catalyzed by dendrimeric ligands and monomer and branch models.

Rhodium complexes with chiral monodentate phosphoramidites provide excellent catalytic activity and selectivity in hydrogenation reactions. However, their use as catalysts in the [2+2+2] cycloaddition reaction is scarce. In collaboration with Prof. Anne Marie Caminade and her group at the Laboratoire de Chimie de Coordination in Tolouse, we decided to attach phosphoramidite ligands as end-goups in phosphorus dendrimers and test them in the [2+2+2] cycloaddition of three alkynes. Phosphoramidite-capped dendrimers of generations 1 to 3 were thus prepared (**G1**, **G2**, and **G3**, Scheme 21) and the catalytic activity of their in situ generated rhodium complexes tested for the cycloaddition between N-tosyl tethered 1,5-diyne **28a** and phenylacetylene (Scheme 22) [46]. In comparing the catalytic activity of the dendrimers with commercially available (S)-MonoPhos and the model of the corresponding monomeric ligand (**M** in Scheme 21) (while maintaining an equal Rh/phosphoramidite ratio and catalytic load in all the tests), it was evident that the catalytic activity was considerably enhanced for dendrimeric ligands **G1**, **G2**, and **G3**. Furthermore, the catalytic system with the third-generation ligand **G3** could be recovered by precipitation and filtration and reused up to three times without a reduction in the yield.

We then proceeded to check the capacity for the enantiodiscrimination of the catalytic system in a reaction that generates a biphenyl compound with axial chirality. We thus tested dendrimeric ligands **G1** to **G3** in the [2+2+2] cycloaddition between N-tosyl tethered 1,6-diyne **28a** and 2-methoxynaphthalene alkynyl derivative **72**, and compared them again with (S)-MonoPhos and monomer model **M** (Scheme 22). As in the previous case, there was a clear dendrimer effect on the activity, but even more interestingly, in the enantioselectivity as well. The catalyst could again be recovered and reused up to three times without a noticeable decrease in either the activity or the enantioselectivity. In order to determine if the enhanced enantiodiscrimination arose from a cooperative effect of only two proximal centers, the catalytic activity of the branch model **B** (Scheme 21) was also evaluated. Surprisingly, both the yield and the enantioselectivity decreased; thus, we postulate that the dendrimeric effect comes from the packing of a large number of chiral ligands in close proximity in the dendrimeric structure.

### 2.5. Combinations of Rh Complexes with N-Heterocyclic Carbene (NHC) Ligands

**Scheme 23:**
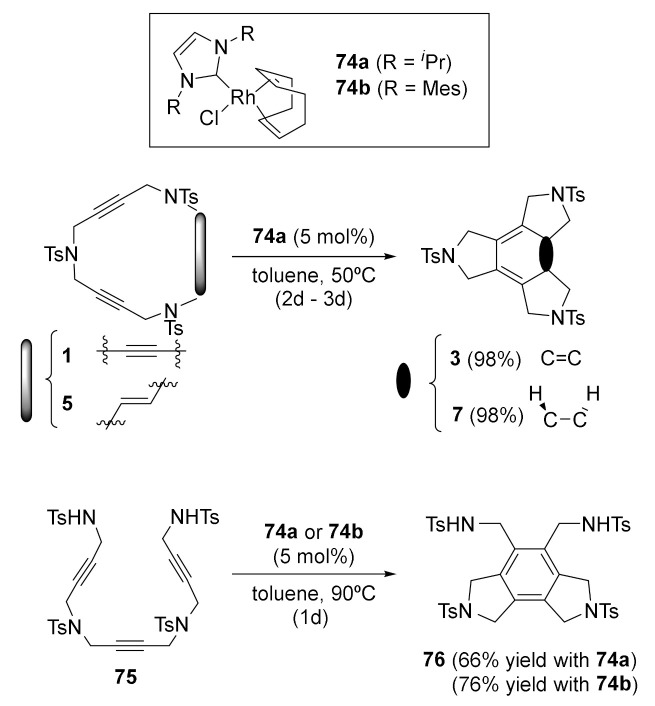
[2+2+2] The intramolecular cycloadditions with Rh-NHC complexes **74a,b**.

Phosphine-stabilized rhodium complexes, whether Wilkinson’s catalyst or combinations of [Rh(cod)_2_]BF_4_ and biphosphines, represent the most common source of rhodium catalysts for these [2+2+2] cycloaddition reactions. However, phosphine ligands are prone to oxidation and the corresponding phosphine oxides formed at the end of the process sometimes hamper product purification. With the aim of exploring topologically new catalytic systems, we wanted to test the ability of readily available rhodium complexes stabilized by N-heterocyclic carbene ligands as catalysts for the [2+2+2] cycloaddition reaction [47] (Scheme 23).

Two carbene–Rh complexes, **74a** and **74b,** were tested in the cycloisomerization reaction of triyne and enediyne macrocycles **1** and **5** and also in the open-chain analogue **75**. The carbene–rhodium complexes were found to be effective in the cycloisomerization process, with the cycloadducts being obtained in excellent yields. Morover, the new complexes proved to be robust enough to permit the reaction to be run under aerobic conditions.

**Scheme 24:**
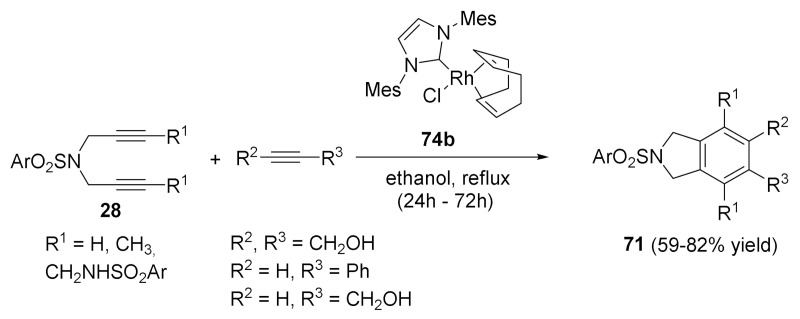
[2+2+2] The intermolecular cycloadditions with the Rh-NHC complex **74b**.

The activity of the Rh–carbene complex **74b** was also examined in the partially intramolecular version of the [2+2+2] cycloaddition reactions. Several N-tosyl tethered diynes **28** were reacted with different acetylenes to afford good to excellent yields of the cycloaddition products **71** in refluxing ethanol (Scheme 24).

As NHC ligands showed good activity in the [2+2+2] cycloaddition reactions, we wanted to again look at the recovery and reuse of the catalytic system, which is an important aspect from an economical and environmental point of view. To this end, we established a collaboration with Prof. Roser Pleixats from the Department of Chemistry at the Universitat Autònoma de Barcelona, whose expertise in hybrid silica materials promted us to prepare rhodium-NHC hybrid silica materials, either by sol-gel or grafting processes, and test them as a recoverable catalysts in cycloaddition reactions [48]. Among many inorganic solids, polymeric silicon oxide is one of the most frequently used catalyst supports, due to its chemical, thermal and mechanical stability. Therefore, we decided to prepare imidazolium salts, conveniently functionalized with trialkoxysilyl groups either in the carbon backbone or in the nitrogen atom, in order to coordinate them with rhodium and incorporate the resulting complexes into hybrid silica materials. Finally, we wanted to test them as recoverable catalysts in [2+2+2] cycloaddition reactions. We show here the preparation of the hybrid material, as well as the catalytic results of the most active material, as an illustrative example.

**Scheme 25:**
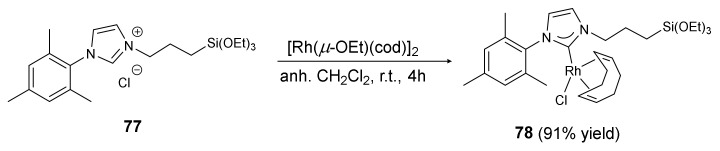
The synthesis of Rh-NHC complex **78**.

Rh-complex **78** was prepared using a previously described method [49], mixing [Rh(μ-OEt)(cod)]_2_ with silylated imidazolium salt **77** (Scheme 25).

**Scheme 26:**
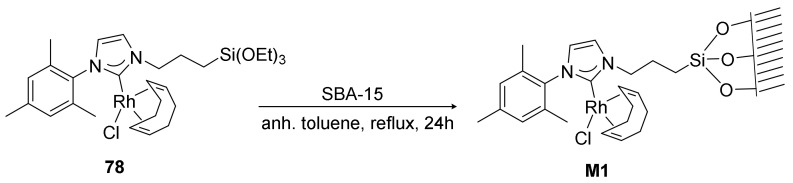
The synthesis of the hybrid silica material **M1**.

A new material, **M1**, was obtained by grafting complex **78** to the mesostructured silica SBA-15 in refluxing anhydrous toluene under nitrogen atmosphere for 24 h (Scheme 26). The new material was characterized by solid state ^29^Si NMR, N_2_-sorption measurements, thermogravimetric analysis (TGA), and elemental analysis, and the amount of rhodium was determined using inductively coupled plasma (ICP) analysis.

**Scheme 27:**
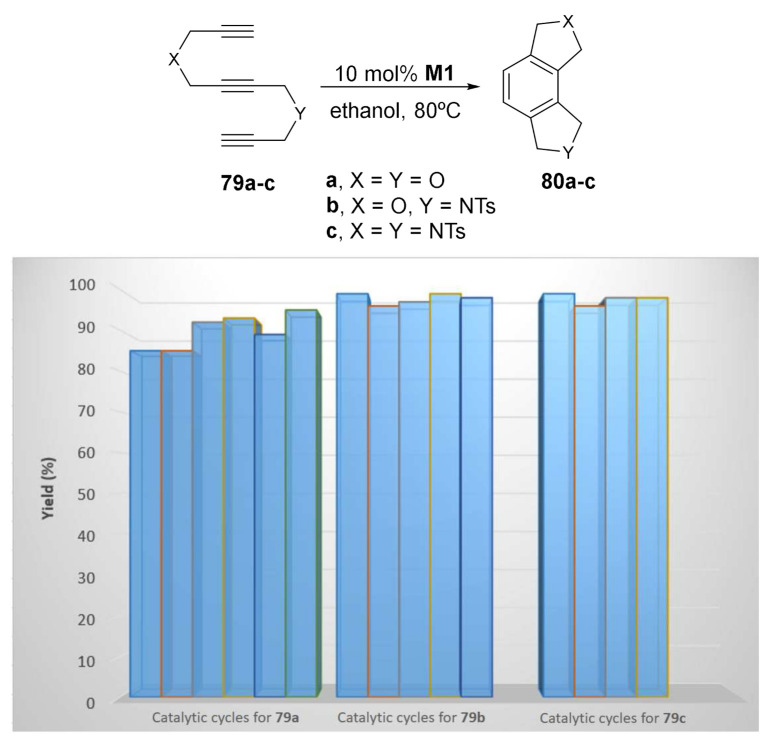
The catalytic performance of the hybrid-silica material **M1**.

The [2+2+2] cycloaddition reaction of the tethered triynes **79** was tested with material **M1**. The cycloaddition was optimized by testing several solvents and reaction temperatures. Ethanol at 80 °C provided the most efficient conditions. For all three enediynes, **M1** could be reused, although longer reaction times were required (Scheme 27).

The amount of rhodium leaching was determined by ICP analysis. After complete conversion of **79** to **80**, the reaction mixture was filtered and the rhodium content in the concentrated final product was 9140 ppm for **M1**, which corresponds to metal losses of 12.15%, with respect to the initial amount of rhodium added as the catalyst. The high values of rhodium present in the product were consistent with the results obtained when hot-filtration tests were performed, as they demonstrated that the catalytic activity partially follows a homogeneous pathway for the grafted material.

**Scheme 28:**
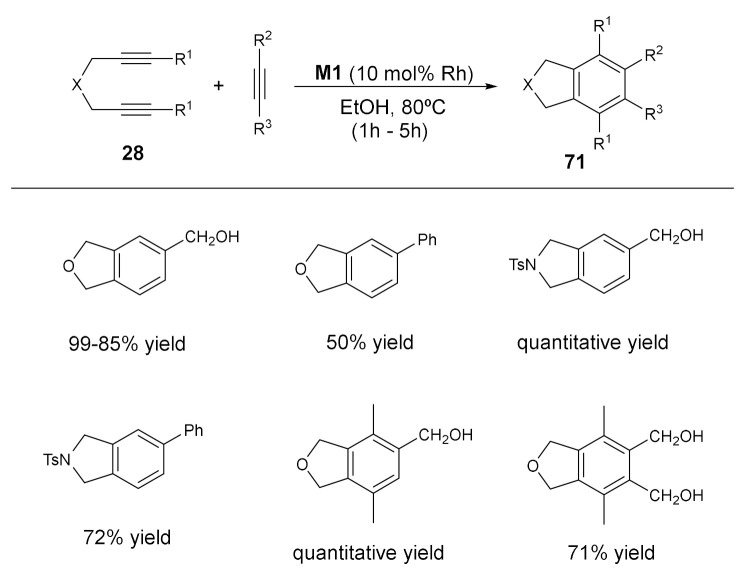
The catalytic performance of the hybrid-silica material **M1**.

We then tested the catalytic activity of material **M1** in the partial intramolecular version of the [2+2+2] cycloaddition reaction between several diynes **28** and monoalkynes. We used the same optimized reaction conditions as for the cycloaddition of triynes. Both terminal and non-terminal diynes with different tethers were active in this process. However, with non-terminal diynes, it was necessary to heat the reaction at 110 °C using n-BuOH as the solvent, as they are less reactive than terminal diynes. Both monosubstituted and disubstituted alkynes gave good yields of the cycloadducts. All cycloadducts were obtained with good purity after simple filtration of the catalyst and evaporation of the solvent (Scheme 28).

## 3. Summary

In this personal account, we have described all the aspects of the fascinating rhodium-catalyzed [2+2+2] cycloaddition reactions that we have studied in depth. While rhodium complexes were always the catalyst of choice, we explored different Rh-based catalytic systems to optimize the different reactions that we observed.

After initial studies in which the reaction was performed inside a triyne macrocycle to afford tetracyclic benzene derivatives, we went on to study several aspects of this highly efficient and atom-economic reaction. Through collaborations with different groups, it was determined that various types of catalytic systems are active in the reaction, including recyclable hybrid silica NHCs (collaboration with Prof. R. Pleixats, Universitat Autònoma de Barcelona), stereoselective P-stereogenic iminophosphoranes (collaboration with Prof. A. Riera, University of Barcelona), and phosphoramidite-decorated dendrimers that showed an unprecedented dendritic effect on enantiodiscrimination (collaboration with Dr. A.M. Caminade, LCC-Toulouse). A second focus of interest was the study of the chemo-, regio- and stereoselectivity of the process by introducing alkenes, and especially allenes, into the reaction. Some of these studies were focused on selectively obtaining polyfunctionalized compounds such as 2,6-naphthyridine scaffolds and densely functionalized cyclohexadiene scaffolds, but most of them where devised to gain fundamental mechanistic knowledge of the reaction. In these mechanistic works, experimental and theoretical tools were used in an intertwined manner to evaluate the electronic effects on the trimerization of phenylacetylenes, the feasibility of the [2+2+2] cycloaddition of C_60_ and two acetylene molecules in the presence of Wilkinson’s catalysts, and the chemoselectivity of different unsaturations in the reaction. More specific details of the mechanism were also evaluated, such as the structure of the third intermediate, which was studied by ESI-MS and DFT calculations, the kinetics of the two main steps of the mechanism, which were studied by cyclic voltammetry (this study was done in a collaboration with Prof. Jutand from Paris), and the possibility of using a chiral induction strategy, which was experimentally evaluated in substrates containing two allenes. We have also reviewed various aspects of the transition metal-catalyzed [2+2+2] cycloaddition reaction, such as the use of allenes reagents, the chiral induction strategies used, and most recently, the mechanistic aspects.

## Figures and Tables

**Table 1:**
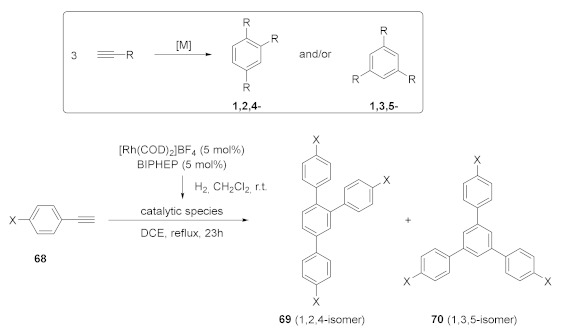
[Rh(cod)_2_]BF_4_/BIPHEP-catalyzed [2+2+2] cycloaddition of the phenylacetylene derivatives **68**.

X	σ-Para	Yield (%) ^1^/Ratio (69:70) ^2^
NO_2_	0.78	38/100:9
F	0.06	36/92:08
H	0	62/96:04
Me	−0.17	96/95:05
^t^Bu	−0.20	92/81:19
OMe	−0.27	65/94:06
NMe_2_	−0.83	33/59:41

^1^ Isolated yield. ^2^ The ratio was determined by ^1^H NMR of the crude mixture.
